# Non-accidental Trauma as an Example of an Underestimated Problem in Pediatrics: Maltreated Child Syndrome and Shaken Baby Syndrome

**DOI:** 10.7759/cureus.86738

**Published:** 2025-06-25

**Authors:** Katarzyna Kubińska, Magdalena Zgoda-Aleksandrowicz, Anita Franczak-Young, Agata Hałabuda, Stanisław Kwiatkowski

**Affiliations:** 1 Department of Medicine, Jagiellonian University Medical College, Kraków, POL; 2 Department of Pediatric Neurosurgery, Institute of Pediatrics, Jagiellonian University Medical College, Kraków, POL; 3 Department of Radiology, Institute of Pediatrics, Jagiellonian University Medical College, Kraków, POL

**Keywords:** abusive head trauma, child abuse, maltreated child syndrome, non-accidental trauma, shaken baby syndrome

## Abstract

Injuries, including both accidental and non-accidental trauma, represent a common reason for hospitalization in the pediatric population. The impact of violence in the caregiver-child relationship is an important and often underestimated problem in pediatrics. Child abuse can present itself in various forms, including both psychological and physical manifestations. This study details the maltreated child syndrome and the shaken baby syndrome, also known as abusive head trauma. Non-accidental trauma presents with a broad spectrum of neurological symptoms, along with multiple injuries at different stages of healing and potential indicators of shaking. A two-month-old male infant presented with body spasms, subsequently accompanied by muscle flaccidity, following an accident of unclear mechanism. Imaging studies revealed bilateral subdural hematohygroma with acute hemorrhage, multiple fractures in various stages of healing, and retinal hemorrhage. Clinical, psychological, and social findings were consistent with shaken baby syndrome and maltreated child syndrome, suggesting non-accidental trauma. Beyond the current injuries, it is significant to be aware of the long-term effects of violence, particularly psychological and social aspects related to neurological damage. The role of medical staff is not only to provide medical help but also to ensure the child’s safety and prevent a negative impact on their psychosocial development. Knowing how to recognize the signs of child abuse is relevant.

## Introduction

Despite the phenomenon of child abuse having a long and inglorious history, it still remains underdiagnosed, and its effects are difficult to detect and prevent. The literature defines non-accidental trauma (NAT) as injury where the reported mechanism is unclear or inconsistent with the clinical findings, commonly resulting from acts like beating, shaking, or sexual abuse [1,2]. All known cases of maltreated child syndrome (MCS) can be regarded as a spectrum of disorders resulting from subjecting the child to various types of aggression. Symptoms may vary depending on the mechanism of the violence. Victims most often present multiple injuries at different stages of healing, with inconsistencies between the reported mechanism and nature of injuries [3]. The typical results of falls should raise suspicion in children who are not yet mobile [2].

Abusive head trauma (AHT) stands out as the leading cause of non-natural death in infancy, and the most common mechanism of AHT among infants is shaking [1]. Shaken baby syndrome (SBS) is described by the following triad: subdural hematoma (SDH), brain swelling, and retinal hemorrhage (RH) [1,4-6]. Rib fractures and bruising of the arms and chest resulting from the child being held tightly during shaking may also be observed [1,2]. Recognizing the subtle signs of child abuse is key to preventing the physical and psychological consequences of violence.

## Case presentation

A two-month-old male infant was admitted to the emergency department due to an episode of generalized seizure, subsequently accompanied by muscle flaccidity, which occurred after an injury. According to the mother’s narrative, a week ago, the older son fell on the baby’s crib. Immediately after the accident, the parents reported to the emergency department. Upon initial examination by the attending physician, no neurological deficits, loss of consciousness, or signs of epileptic activity were identified. A fracture of the left humerus with displacement was diagnosed, and a splint was applied to the affected limb.

Following the injury, the mother reported a progressive reluctance to feed, as well as increased regurgitation and vomiting. Given the context, the diagnosis was extended towards a possible craniocerebral trauma. Besides scalp bruising, SDH was detected on transfontanelle ultrasonography. Consequently, the patient was referred to a specialist center for a CT scan of the head under general anesthesia.

On admission to the department of pediatric neurosurgery, the infant was in stable condition, conscious, and demonstrated appropriate responsiveness. Physical examination revealed no alarming symptoms. The splint on the left upper limb was present. Breastfeeding appeared unimpaired. The patient did not require urgent neurosurgical intervention. There was a family history of hematological diseases (mother’s thrombophilia, brother’s PAI-1 deficiency). No pathologies within the CNS were found during pregnancy.

**Figure 1:**
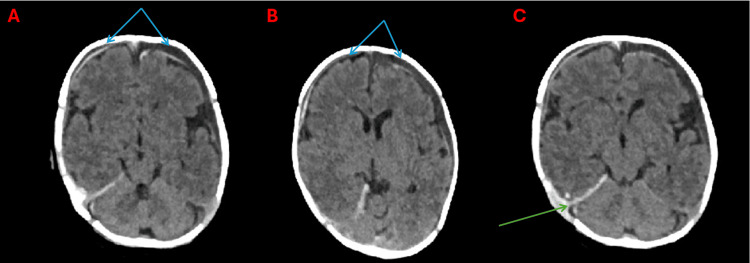
Non-contrast head CT, axial sections at three levels, brain window. Bilateral subdural hematohygromas in the frontal region (A and B, blue arrows). Bleeding along the right side of the cerebellar tentorium (C, green arrow).

On the day of admission, a non-enhanced CT scan of the head was performed under general anesthetic. The examination revealed bilateral subdural hematohygroma with signs of acute bleeding in the frontotemporal regions and additionally in the left parietal region. It also showed bleeding along the attachment of the cerebellar tentorium on the right side and pericerebral hematoma at the level of the dilated lambdoid suture on the right side (Figures 1A-1C).

**Figure 2:**
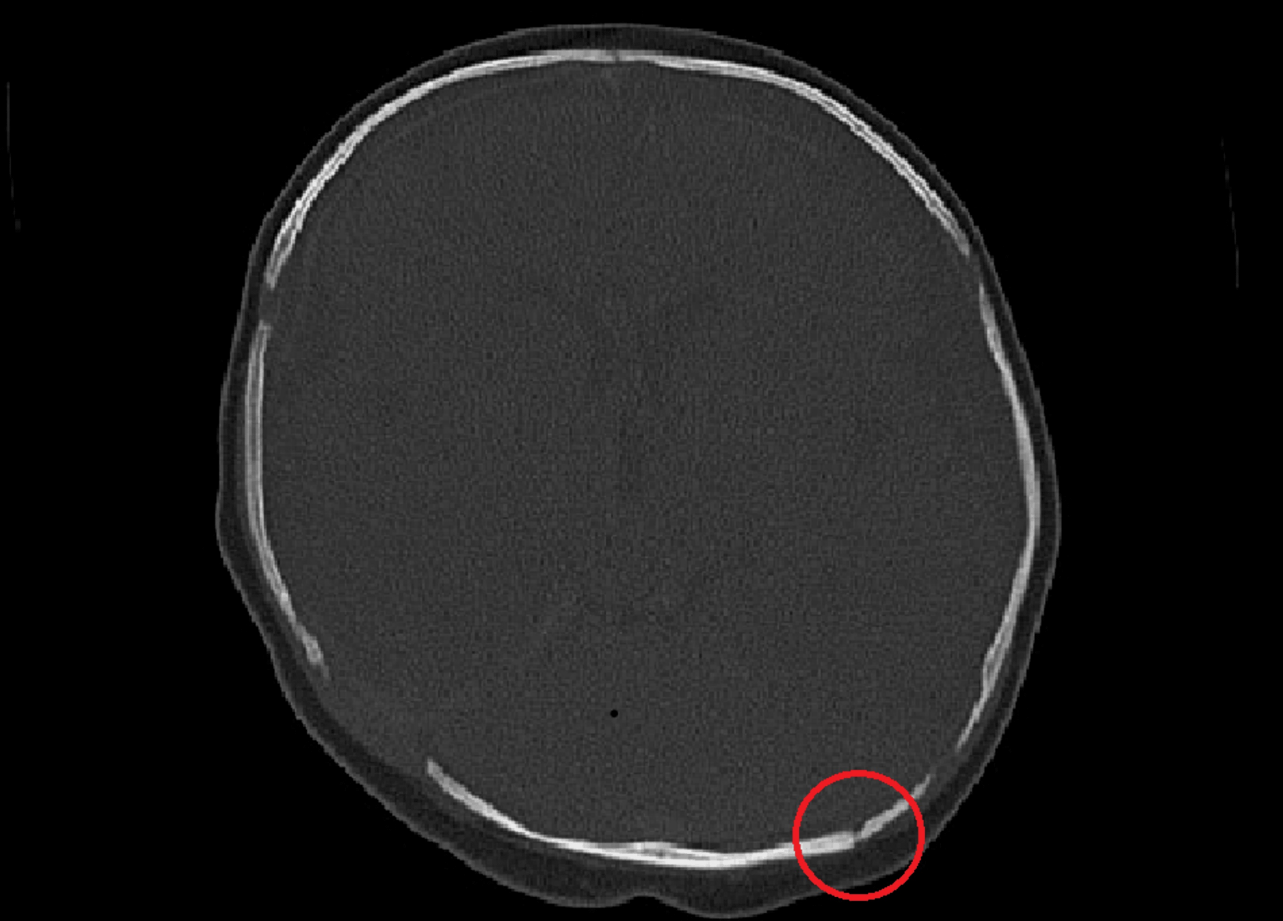
Non-contrast head CT, axial section, bone window. Squamous part of the occipital bone: fracture line on the left side (red circle); asymmetry of the lambdoid suture.

Additionally, fracture of the squamous part of the occipital bone on the left side was described, along with asymmetry of the lambdoid suture (wider on the right side), likely due to traumatic diastasis (Figure 2). A control CT scan performed on the fourth day of hospitalization showed still visible SDH and hematohygroma in the hemolysis phase. Surgical treatment was deemed unnecessary. Conservative treatment was indicated.

**Figure 3:**
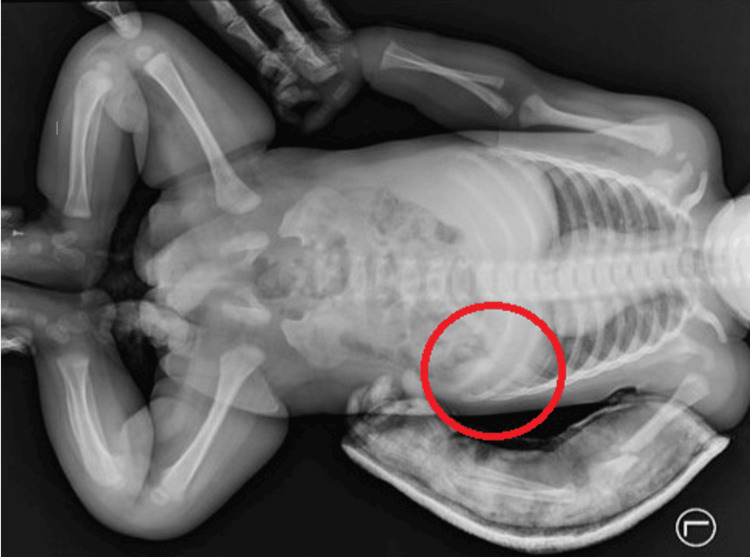
The condition following fractures of the 10th and 11th ribs on the left side in the posterior segment, currently in the healing phase, with visible localized periosteal thickening. The ninth left rib in the posterior paravertebral region shows localized thickening and periosteal layering, also raising suspicion of a fracture in the healing phase (red circle).

It was decided to extend diagnostics with a babygram - a whole-body X-ray image performed in special cases of congenital diseases and when the etiology of injuries may not be accidental. In this case, the presence of multiple injuries at various stages of healing indicated the need for a babygram. The examination confirmed a recent fracture with displacement of bone fragments of the left humerus (splint present), along with previously sustained fractures of the ninth, 10th, and 11th ribs, currently in the healing phase (Figure 3).

Due to the family history of thrombophilia in the mother, it was decided to consult the boy with a hematologist. Moderate thrombocytopenia, decreased levels of factors IX and V, and increased D-dimers were observed, without other pathological results. An ophthalmological examination revealed a blurred optic disc border and retinal hemorrhage (RH) in the right eye.

A regression in the child's psychological development was observed. According to the mother, he smiled spontaneously before the incident, but this was no longer observed during the hospital examination. He did not respond to calling and was lethargic. In the neuropsychologist’s opinion, the randomness of the events could be ruled out, and the boy’s parents should be suspected of abuse. The mother showed limited interest in the child's medical prognosis, focusing mainly on the discharge date. No signs of aggressive behavior were observed during her stay in the ward. The father's presence was not recorded, and contact with him could not be established.

Signs of abuse were clearly identified, indicating both shaken baby syndrome (SBS) and other injuries (MCS) linked to intentional violence. The social-legal department was notified and, consequently, the Municipal Social Welfare Center was informed about the child's situation. After 11 days of hospitalization, the boy was discharged from the hospital in good general condition. Although she was advised to attend follow-up appointments, the mother did not comply.

## Discussion

Diagnosing NAT is a considerable challenge in pediatric healthcare due to its subtle nature and the difficulties in obtaining accurate statistics. AHT can lead to serious long-term disabilities and even fatalities among affected children, especially infants [1,5,7]. Therefore, clinicians need to differentiate between accidental (traumatic) and non-accidental causes of head injuries [5].

The World Health Organization, in the report of the Consultation on Child Abuse Prevention (Geneva, 1999), defines child abuse as "all forms of physical and/or emotional ill-treatment, sexual abuse, neglect or negligent treatment, or commercial or other exploitation, resulting in actual or potential harm to the child’s health, survival, development or dignity in the context of a relationship of responsibility, trust or power". This definition is further expanded in the World Report on Violence and Health (Geneva, 2002), which notes that neglect or failure to protect children from exposure to violence also constitutes abuse. Passivity towards violence also contributes to harm to children. Therefore, being attentive to subtle signs of aggression is key to protecting the health and lives of young patients. The tools necessary to identify NAT do not require significant time or financial outlays. These primarily include an interview and a physical examination, along with transfontanelle ultrasonography and fundoscopy, which are essential for diagnosing AHT [1]. A discrepancy between the clinical condition of the child with the reported mechanism of injury and inconsistencies in the caregivers' narrative constitute an important alarm signal.

SDH with or without a skull fracture is the most common finding in AHT victims [1,2]. Extravasation of blood into the subdural space is caused by whiplash injuries, in which the rapid forward and backward movement of the head without local trauma causes rupture of the bridging veins. Infants are particularly susceptible to this type of injury due to the imbalance between the weight of the head and the strength of the neck muscles [1]. Initially, SDH will not cause intracranial pressure symptoms because the extravasated blood mixes with the cerebrospinal fluid and accumulates in the longitudinal fissure and over the cerebral hemispheres. After exhaustion of adaptive mechanisms, after about 14 days, irritability, drowsiness, inconsolable crying, stiff neck, vomiting, and seizures may occur [8]. Fundoscopic examinations may reveal RH resulting from shaking, which can cause significant deterioration of vision and even blindness. Damage to the CNS sustained during violence prevents the child from further developing properly [6]. The simultaneous occurrence of SDH and RH provides a pathognomonic presentation for diagnosing SBS. However, it is critical to acknowledge that the presence of only one symptom does not rule out the diagnosis [1].

Long-term outcomes of NAT extend beyond immediate physical injuries, leading to profound cognitive impairments and difficulties in social functioning. Children with a history of AHT often exhibit diminished cognitive abilities due to the damage sustained in the grey matter of the temporal and frontal lobes. It may lead to a gradual deterioration of the child's IQ. Changes observed in the limbic system may contribute to emotional regulation difficulties, which in turn translate into impaired function in society [9]. Victims of violence often have lower results in school and require specialized educational approaches tailored to their unique needs. The assessment of long-term effects is limited by the fact that caregivers who commit violence often do not report for follow-up visits [7].

Notably, while some acts of violence against children are perpetrated with intentional harm, many incidents are not intentional. SBS may arise from unpreparedness for the role of a parent in the psychological and social sense, as well as fatigue and frustration, which is most often (60%) triggered by a child’s inconsolable crying [7]. A significant deterioration in functioning may occur at the time of creating a relationship with the child in the case of people with certain personality traits that predispose to such maladaptive parenting behavior - these are not necessarily psychopathic or sociopathic personalities [3]. Furthermore, exposure to aggression in childhood is also a significant risk factor for becoming a victim or perpetrator in adulthood due to the repetition of childhood patterns [3]. A parent who shakes a crying child, even briefly, may not realize that this seemingly calming act can cause permanent health damage. [7]. It could result from a lack of knowledge and a momentary loss of control over his own emotions. The solution is to attach greater importance to increased training and education for caregivers to mitigate risks and promote healthier parenting practices. The multifaceted nature of AHT requires a concerted effort from healthcare professionals, educators, and communities to adequately protect and support affected children.

## Conclusions

Child abuse is a serious issue that can affect children at any age and often presents with subtle or non-specific clinical signs. This fact should motivate clinicians to be vigilant when taking the medical history and conducting physical examinations. Particular attention should be paid to the presence of multiple injuries at various stages of healing, especially when the reported mechanism is unclear or inconsistent with the nature of the injuries. In such cases, imaging tests (such as a babygram, ultrasound, or CT scan), as well as ophthalmological and psychological consultations, should be promptly performed.

The role of the medical team extends beyond the diagnosis and treatment of physical injuries. Ensuring the safety of the child and minimizing the risk of further harm must be prioritized through early recognition, proper documentation, and collaboration with child protection services. A multidisciplinary approach is essential, not only for effective clinical management but also for the long-term psychological and social well-being of the child. Continued education and awareness among clinicians are critical in improving detection and response to non-accidental trauma.

## References

[1] Oruç M, Dündar AS, Okumuş H, Görmez M, Şamdancı ET, Celbiş O (2021). Shaken baby syndrome resulting in death: a case series. Turk J Pediatr.

[2] Biswas A, Shroff MM (2021). Abusive head trauma: Canadian and global perspectives. Pediatr Radiol.

[3] Kempe CH, Silverman FN, Steele BF, Droegemueller W, Silver HK (1962). The battered-child syndrome. JAMA.

[4] Vinchon M, Noulé N, Karnoub MA (2022). The legal challenges to the diagnosis of shaken baby syndrome or how to counter 12 common fake news. Childs Nerv Syst.

[5] Vinchon M, Di Rocco F (2022). Abusive head injuries in infants: from founders to denialism and beyond. Childs Nerv Syst.

[6] Kelly JP, Feldman KW, Weiss A (2024). Optical coherence tomography and visual outcomes in pediatric abusive head trauma. Retin Cases Brief Rep.

[7] Chang HY, Chang YC, Chang YT, Chen YW, Wu PY, Feng JY (2024). The effectiveness of parenting programs in preventing abusive head trauma: a systematic review and meta-analysis. Trauma Violence Abuse.

[8] Smith EB, Lee JK, Vavilala MS, Lee SA (2019). Pediatric traumatic brain injury and associated topics: an overview of abusive head trauma, nonaccidental trauma, and sports concussions. Anesthesiol Clin.

[9] Tomoda A, Nishitani S, Takiguchi S, Fujisawa TX, Sugiyama T, Teicher MH (2024). The neurobiological effects of childhood maltreatment on brain structure, function, and attachment. Eur Arch Psychiatry Clin Neurosci.

